# Community Readiness Model Applied in Older Adults for the Promotion of Digital Cognitive Training: Mixed Methods Feasibility Study

**DOI:** 10.2196/69434

**Published:** 2026-02-19

**Authors:** Cíntia Monteiro Carvalho, Bruno Costa Poltronieri, Beatriz Pereira da Silva Lima, Rafaela Guilherme Ferreira, Maria Eduarda Alves Reis, Christine FitzGerald, Brian Lawlor, Rogério Panizzutti

**Affiliations:** 1 Instituto de Psiquiatria Universidade Federal do Rio de Janeiro Rio de Janeiro Brazil; 2 Instituto de Ciências Biomédicas Universidade Federal do Rio de Janeiro Rio de Janeiro Brazil; 3 Instituto Federal de Educação Ciência e Tecnologia do Rio de Janeiro Rio de Janeiro Brazil; 4 Global Brain Health Institute Trinity College Dublin Dublin Ireland; 5 Ageing Research Centre University of Limerick Limerick Ireland

**Keywords:** healthy aging, cognitive training, internet-based interventions, behavior change, community participation

## Abstract

**Background:**

Digital cognitive training, which involves structured, digital exercises designed to enhance cognitive functions, has shown potential benefits for older adults. While digital cognitive training has shown potential benefits for older adults, successfully incorporating it into their daily routines remains a challenge. Community readiness refers to the group’s ability and capacity for a behavior change to be more effective and sustainable. In this study, readiness specifically refers to the community’s preparedness to engage in and sustain digital cognitive training.

**Objective:**

This study aimed to evaluate the feasibility of the Community Readiness Model (CRM) to identify facilitators and barriers in implementing training and applying supportive strategies to increase readiness.

**Methods:**

This mixed methods feasibility study was conducted as part of a stepped-wedge randomized controlled trial examining the effects of digital cognitive training on functionality and cognition in older adults. Fifty-six participants were recruited from a nursing home and the community and were allocated to one of two training protocols: one group played digital leisure games for 10 hours followed by 10 hours of digital cognitive training, while the other group completed 20 hours of digital cognitive training. Readiness levels were assessed using CRM through semistructured individual interviews conducted before the intervention. Additionally, the CRM was administered to a sample of stakeholders to evaluate community-level readiness. Following the implementation of strategies aimed at enhancing readiness, as well as the delivery of the digital cognitive training, the interviews were readministered to evaluate changes in readiness levels.

**Results:**

Before the training, participants demonstrated low levels of readiness, with a median CRM total score of 3.6 (IQR 2.2-4.6) in the 10-hour training group and 3.7 (IQR 2.7-4.3) in the 20-hour group. To address this, several strategies were implemented: dissemination of psychoeducational content via social media, distribution of cognitive exercise guides, stakeholder engagement to raise project awareness, and provision of necessary devices along with comprehensive support throughout the training. Following the implementation of these strategies and the training sessions, a significant increase in readiness was observed. Both groups achieved a median CRM total score of 6 (IQR 5-7). Participants identified key facilitators as interest in the training, noticeable cognitive improvements, team support, motivation to continue, the challenge presented by the training, and overall satisfaction. The primary barrier reported was difficulty using the technology.

**Conclusions:**

The CRM demonstrates strong feasibility as a tool for identifying facilitators and barriers that can inform strategies to enhance readiness for digital cognitive training in older adults. The observed increase in readiness scores following the implementation of targeted strategies highlights the CRM’s potential to guide the development of effective, supportive interventions. These findings emphasize the importance of addressing technological challenges while harnessing motivating factors to promote the successful adoption of digital cognitive training within this population.

## Introduction

Aging is associated with a progressive decline in several brain functions including sensory perception, attention, memory, and motor control, which is termed “age-related cognitive decline.” Age-related cognitive decline is associated with a poorer quality of life, less independence, and a higher cost for the health system [1]. Therefore, investing in preventive measures to mitigate cognitive decline is necessary. Based on robust research that has elucidated the fundamental principles of brain plasticity, it is now well established that dedicated behavioral training can bring about substantial improvement in cognitive function or recovery [2-6].

Digital cognitive training has emerged as a promising intervention to enhance cognition in older adults. By engaging in these training programs, individuals can either increase their neural capacity or improve the efficiency of existing brain resources [3]. This approach generally focuses on improving general cognitive processes such as processing speed, attention, and working memory, with the premise that these improvements will transfer to real-world cognitive gains [7]. Systematic reviews and meta-analyses of digital cognitive training for older adults have concluded that these training interventions can be effective for older adults and thus should be recommended [8-11].

While the benefits associated with cognitive training for older adults are well recognized, a challenge lies in effectively implementing digital cognitive training in their lives. Previous studies that provided cognitive training to older adults were conducted under the constraints of research protocols and did not consider the resources available to the participants nor the effective behavioral and societal changes needed to ensure engagement with users. Our previous experience with clinical trials and other studies using digital cognitive training has revealed that several training-related factors affect engagement, including personal support, location, attitudes toward new learning and technology, and communication between the users [12-16].

The Community Readiness Model (CRM) is a theory-based strategic model used to assess and build community capacity to tackle social issues. The CRM allows the community’s influence and knowledge to assume a central role in the application of the model, allowing researchers to meet communities where they are and on their own terms [17]. The concept of CRM revolves around the idea that starting a new program or intervention is only appropriate when the community is ready. For community interventions or programs to be successful, a community must be consistent with its awareness of the problem and its readiness for change [16,18]. The concept of community adopted in this study focuses on a community of interest defined by shared interests regardless of individuals’ location or social group [19,20]. By tailoring interventions to a community’s specific needs and readiness, CRM not only identifies facilitators and barriers but also promotes skill development among community members. This collaborative approach is essential for fostering sustainable change and empowering individuals to tackle the challenges they face. Engaging the community in this way ensures that solutions are relevant and supported, ultimately leading to more successful outcomes [21].

Previous applications of the CRM were effective in addressing a variety of health issues, such as health service delivery [22,23], substance use [24], childhood obesity [25], and mental health awareness [21]. On the other hand, for the design and implementation of community-based interventions, the involvement of stakeholders (eg, health promotion professionals) is important, since they can help ensure that the intervention planning is suitable given the real conditions [26,27].

Given the complexity of promoting affective engagement in digital cognitive training—an essential factor for effectively integrating such interventions into the daily routines of older adults [28]—we chose to use the CRM as a framework to support this behavior change. In the context of our study, readiness refers to the community’s preparedness to adopt and support training initiatives. The CRM offers valuable insights into both behavioral factors (eg, familiarity with technological tools) and personal factors (eg, an individual’s interest in participating in a specific intervention). Unlike other models, the CRM has the distinct advantage of incorporating a community-based perspective, providing a comprehensive assessment of a community’s readiness. These features are critical for tailoring strategies that align with the community’s current level of readiness, thereby enhancing the likelihood of implementing an effective and sustainable intervention.

We hypothesized that the CRM can be a useful and viable tool to identify barriers and facilitators in the training, which can then be used to develop strategies to increase community readiness for digital cognitive training. To examine this hypothesis, the primary objective of this study was to provide evidence for the feasibility of using the CRM to evaluate the readiness of older adults undergoing digital cognitive training and identify facilitators and barriers associated with the training. The secondary objective was to implement strategies aimed at enhancing older adults’ readiness for training and to evaluate changes in their readiness levels after completing a digital cognitive training program. To achieve the proposed objectives, we applied the CRM to a sample of older adults before and after the implementation of the training. The model was also applied to a sample of stakeholders. We used the results of the preintervention quantitative and qualitative analysis to develop and implement strategies to increase the readiness level of older adults.

## Methods

### Design

This mixed methods feasibility study is part of an ongoing double-blind, stepped-wedge randomized controlled trial investigating the effects of different doses (10 or 20 hours) of digital cognitive training on cognitive function and functionality in older adults. We hypothesized that participants who received 20 hours of digital cognitive training would exhibit sustained improvements in both cognitive function and functionality. All participants underwent a comprehensive initial assessment, including clinical history, as well as assessments of cognition, functionality, and symptoms of anxiety and depression. They were randomized using a hierarchical stratification by gender, age, years of education, Montreal Cognitive Assessment test score, and the Technology Activities of Daily Living Questionnaire score. They were allocated to one of two training protocols: one group played digital games for leisure for 10 hours followed by 10 hours of digital cognitive training, while the other group engaged in 20 hours of digital cognitive training. The stepped-wedge randomized controlled trial involves random and sequential crossover of groups from control to intervention until all groups are exposed to the intervention [29]. This allowed all participants to perform digital cognitive training during the study. Participants and researchers were blinded throughout the study.

### Ethical Considerations

This study was conducted in accordance with the Declaration of Helsinki and was approved by the University Hospital Clementino Fraga Filho Ethics Committee (CAAE: 82713418.6.0000.5257). All participants (older adults and stakeholders) provided written informed consent prior to participation. Participant privacy was protected through data de-identification and secure data storage, with access restricted to the research team. No financial compensation or incentives were provided.

### Participants and Training Conditions

Participants were recruited by advertising on social media and in a nursing home in Rio de Janeiro, or through referrals from health professionals, between April 2019 and November 2021. We invited older adults aged 60 years or above and included those who (1) provided written informed consent, (2) had normal or corrected vision and hearing, (3) had access to a computer and internet at home, and (4) were native Portuguese speakers and literate. Participants were excluded if they (1) had a diagnosis of dementia, (2) were dependent on activities of daily living, or (3) had major medical conditions that would prevent them from participating in the study.

The training platform used was BrainHQ from Posit Science, Inc, and the exercises were previously selected and aimed to improve executive functions. The exercises for cognitive training were as follows: *divided attention*, *eye for detail*, *target tracker*, *juggle factor*, and *mind-bender*. These exercises dynamically adjusted their speed and stimulus type based on participant performance. As participants improved, task difficulty systematically increased, and an algorithm maintained individual success rates around 85%. This threshold was incorporated into the design of the training program as a means of achieving an optimal balance between cognitive challenge and sustained learner engagement [4]. To enhance motivation, correct responses were rewarded with engaging visual and auditory feedback, alongside the accumulation of stars. After completing the intervention, participants received feedback provided by the BrainHQ platform on their progress in trained cognitive domains.

The leisure games condition served as a control for potential confounding factors such as computer exposure, researcher interaction, and nonspecific cognitive engagement—including attention, executive functions, and motivation—stimulated by graphics-based digital games. The leisure games were commercial online: *Bubble Poke*, *Smarty Bubbles*, *Dominoes Classic*, *Puzzle*, *Find 500 Differences*, *Find the Birds*, *Let’s Clean Up*, and *Crosswords*.

In both groups, participants engaged in 15-minute sessions of 4 predetermined exercises, following the established protocol. They were encouraged to train at least twice a week. The intervention of both groups was accompanied by a video call by a research group member who explained each exercise or game in advance and supported the user by answering questions when necessary.

### Stakeholders’ Participation

Based on previous studies indicating that stakeholder engagement is associated with improved community readiness for facilitating change [30,31], we determined that stakeholder participation was essential for understanding community readiness and identifying potential barriers and facilitators to intervention implementation. Prior to launching the digital cognitive training program, we conducted interviews with 7 stakeholders, including 4 health professionals (a physical therapist, an occupational therapist, a physician, and a speech therapist) and 3 family members of study participants. All stakeholders had direct contact with the participants. Recruitment took place at a nursing home in Rio de Janeiro and through referrals from professionals in the field. Interviews were conducted prior to the intervention, and the CRM was applied at this stage to gather additional insights into participants’ initial level of readiness.

### Community Readiness Model

The CRM was applied during the initial assessment and after participants completed either 10 or 20 hours of training, through semistructured individual interviews conducted via video call. The semistructured interview had 11 questions that allowed participants to talk about access, perceptions, expectations, and their experiences regarding the use of technological resources and digital cognitive training. The questions were developed following the criteria established in the manual proposed by Stanley [32], which assesses the community’s readiness by addressing 5 key dimensions that are crucial to initiate and sustain healthy changes. These dimensions are *knowledge of the issue*, which refers to how much the community knows about the issue; *knowledge of efforts*, which refers to how much the community knows about the programs and activities of digital cognitive training; *community climate*, which reports the community’s attitude toward addressing the issue; *leadership*, which indicates the leadership’s attitude toward addressing the issue; and *resources*, which states how much the resources are being used or could be used to address the issue. The “leadership” dimension was not included in the interview, as the description of this dimension did not apply to the community context.

Each dimension is scored on a scale of 1 to 9, corresponding to a stage. In the initial stage of readiness, “no awareness” (1), the community lacks information about local initiatives, resulting in little awareness of the problem. As the community progresses to “denial/resistance” (2), there is resistance to acknowledging the issue due to a lack of accurate information. Community perception improves slightly in the “vague awareness” stage (3), but knowledge remains superficial. “Preplanning” (4) involves recognizing the concern more concretely, though knowledge and resources are limited. During “preparation” (5), awareness increases, and leadership supports efforts with directed resources. In “initiation” (6), most members have a basic understanding, and leadership plays a central role in planning additional initiatives. Community knowledge solidifies in the “stabilization” stage (7), and leadership ensures long-term viability with continuous resources. “Confirmation/expansion” (8) sees high community knowledge and leadership expanding initiatives, indicating lasting commitment. In the final stage, “high level of community ownership” (9), the community shows substantial knowledge, with active involvement and support from leadership, adjusting resources based on continuous evaluations. Each dimension is scored on a rating scale from 1 to 9, corresponding to these stages.

The CRM interviews were audio-recorded, transcribed, and then analyzed and scored by two independent researchers. The CRM scoring process followed the CRM handbook using anchored rating scales. After establishing a score by each researcher, a consensus was reached on the final score.

### Development of Behavior Change Strategies

We implemented strategies to enhance readiness levels, drawing on each dimension of the CRM and incorporating behavior change techniques (BCTs). BCTs are commonly used to promote adherence to specific interventions [33]. The BCT taxonomy, a widely recognized framework, organizes these techniques into 16 main categories encompassing 93 distinct methods. For instance, it includes techniques such as “graded tasks,” which involve starting with simple, easy-to-perform activities and progressively increasing their difficulty in a manageable way until the desired behavior is successfully achieved [34]. Strategies were informed by pretraining interviews and community feedback, with the aim of ensuring relevance and effectiveness. Implementation occurred concurrently across both groups, with no distinctions made between them.

### Clinical Data and Assessments

We collected clinical and demographic variables from the participants. In addition, we assessed cognition through the application of the Montreal Cognitive Assessment [35] test; to measure functionality, we administered the Technology Activities of Daily Living Questionnaire [36]; to evaluate anxiety symptoms, we used the Geriatric Anxiety Inventory with 20 agree/disagree items [37]; and to measure the symptoms of depression, we used the 15-item version of the Geriatric Depression Scale [38].

### Feasibility Outcomes

The primary results regarding the acceptability of the intervention were adapted from the study by Proctor et al [39].

- Recruitment rate: the sample size of recruited participants followed the manual’s recommendation of conducting at least 6 interviews, with the possibility of more to obtain a comprehensive understanding of the community [32].
- Attendance: overall attendance rate of >60% [40].
- Attrition: retention rate of at least 75% of participants to the follow-up [40].
- Acceptability of intervention: it was assessed through the responses obtained from semistructured interviews using the CRM.

### Data Analysis

The Shapiro-Wilk test was applied to determine data normality. For nonnormally distributed data, nonparametric statistics were used in the analyses. The independent sample *t* test was used to compare the two groups at baseline, the chi-square test was used to assess previous contact with a computer and gender, and the Wilcoxon test was used to verify differences of dimensions between pre- and postintervention. To identify the facilitators and barriers in the baseline and postintervention, we executed the descending hierarchical classification. This method analyzes the lexical context and generates a hierarchical scheme of word classes that are grouped into similarity classes by the chi-square test. High chi-square values characterize strong associations between word and class; we considered words with a *χ*²_1_>3.8, corresponding to *P*<.05, for the class groupings. This analysis uses words in their reduced forms, and a minimum use of 75% of the total text segments (TS) of the original corpus is required [41]. The rate of textual segments below 75% generated by the software suggests a less homogeneous textual corpus, more dispersed concerning the analyzed content, and less representative [42]. From the TS assigned to each of the word classes revealed by the software, the data were analyzed and interpreted by the researchers to identify barriers and facilitators. The general corpus of textual analysis performed at baseline comprised 56 texts, separated into 652 TS, using 497 TS (76.2%). The postintervention analysis comprised 21 texts for the 10-hour training group, separated into 221 TS, using 172 TS (77.8%), and 20 texts for the 20-hour training group, separated into 245 TS, using 185 TS (75.5%). The data that support the findings of this study are available on request from the corresponding author. We performed all quantitative statistical analyses using SPSS software (v. 26.0; IBM Inc) and the qualitative analysis using the IRAMUTEQ software (Université de Toulouse).

## Results

### Feasibility Outcomes

#### Recruitment Rate and Participant Characteristics

A total of 56 participants were recruited, adhering to the criteria outlined in the recruitment rate guidelines. The cohort was predominantly female, with 43 out of 56 participants (77%), and had an average age of 76.98 (SD 7.7) years and 12.77 (SD 5.03) years of education. Table 1 shows the clinical and demographic characteristics of the total sample and by group, and compares the groups concerning age, gender, education, weekly physical activity practice, number of falls in the last year, previous contact with a computer, cognition, and mood. There were no significant differences between the groups that performed 10 or 20 hours of training regarding any demographic or clinical variables.

**Table 1:** Participant characteristics.

			Total (n=56)		Group 10 hours training (n=31)		Group 20 hours training (n=25)		*P* value	
Age (years), mean (SD)			76.98 (7.70)		77.52 (7.91)		76.32 (7.54)		.56	
Female, n (%)			43 (77)		24 (77)		19 (76)		.90	
Education (years), mean (SD)			12.77 (5.03)		12.55 (4.97)		13.04 (5.18)		.72	
Physical activity (times per week), mean (SD)			1.77 (1.89)		2.00 (1.98)		1.48 (1.78)		.31	
Number of falls (past year), mean (SD)			0.29 (0.78)		0.27 (0.94)		0.32 (0.55)		.80	
**Previous contact with computer, n (%)**										.93
	None	13 (23)		7 (23)		6 (24)				
	Low	26 (46)		14 (45)		12 (48)				
	Medium	6 (11)		3 (10)		3 (12)				
	High	11 (20)		7 (23)		4 (16)				
MoCA^a^ score, mean (SD)			20.88 (2.97)		21.10 (3.03)		20.60 (2.94)		.54	
GDS^b^ score, mean (SD)			3.79 (2.54)		3.48 (2.56)		4.16 (2.52)		.32	
GAI^c^ score, mean (SD)			6.46 (4.88)		5.78 (4.52)		7.44 (5.35)		.30	

^a^MoCA: Montreal Cognitive Assessment.

^b^GDS: Geriatric Depression Scale.

^c^GAI: Geriatric Anxiety Inventory.

#### Attrition Rate

In the group that underwent 10 hours of training, 7 participants dropped out of the study during the first 10 hours of games: 4 due to lack of interest and 3 due to health problems, resulting in an attrition rate of 22%. However, none of the participants dropped out during the 10 hours of digital cognitive training. Additionally, we missed data for another 3 participants in this group. While in the group that completed 20 hours of training, 3 participants dropped out of the study. During the first 10 hours of training, 1 participant left due to lack of interest, resulting in an attrition rate of 4%. During the next 10 hours of cognitive training, 1 participant left due to lack of interest and 1 due to health problems, resulting in an attrition rate of 8%. Furthermore, we had missing data for 2 more participants in this group. Missing data resulted from participants’ unavailability for the postintervention interview despite having completed the training.

#### Attendance

Attendance was 100% among participants in both groups.

### Community Readiness Model Score

First, we analyzed the readiness level for both groups before starting the training. Before the training, the participants had a low level of readiness both for the CRM total and for the dimensions separately, being in the “denial/resistance,” “vague awareness,” or “initiation” stages (Table 2).

**Table 2:** Readiness level at baseline.

	Group 10 hours training (n=21)			Group 20 hours training (n=20)	
	Median (IQR)	Stage of readiness	Median (IQR)		Stage of readiness
CRM^a^ total	3.6 (2.2-4.6)	Vague awareness	3.7 (2.7-4.3)		Vague awareness
Knowledge of issue	2.0 (3.0-4.0)	Denial/resistance	2.0 (3.0-4.0)		Denial/resistance
Knowledge of efforts	2.0 (1.0-3.0)	Denial/resistance	2.0 (2.0-3.0)		Denial/resistance
Community climate	3.5 (3.0-4.0)	Vague awareness	3.5 (3.0-4.0)		Vague awareness
Resources	6.0 (3.0-7.0)	Initiation	6.0 (4.0-7.0)		Initiation

^a^CRM: Community Readiness Model.

### Readiness of Stakeholders

To gain a broader understanding of the community’s readiness, we also assessed the readiness of stakeholders and found it to be consistently low in all areas. The stage and readiness level for each dimension were as follows: knowledge of efforts, “initiation” (5.71); knowledge of the subject, “initiation” (5.57); community climate, “preplanning” (4.14); leadership, “vague conscience” (3.28); and resources, “vague conscience” (3.42).

### Facilitators and Barriers Related to Digital Cognitive Training at Baseline

To get closer to the real needs of older adults in the face of digital cognitive training, we went beyond the quantitative analysis and performed qualitative analysis of the interviews to identify the facilitators and barriers at baseline. The content generated by the IRAMUTEQ software was categorized into specific classes and classified as either a facilitator or a barrier. Examples of quotes for each class are described in a later section.

At baseline, the facilitators were the following. (1) “Curiosity” (TS=100/599, 16.7%): refers to the curiosity to know and understand how cognitive training works and the associated benefits. (2) “Search for cognitive stimulation” (TS=103/599, 17.2%): refers to the participants’ search for resources to improve cognitive abilities. (3) “Getting results” (TS=130/599, 21.7%): refers to participants’ interest in training gains because they perceive cognitive difficulties. (4) “Interest in the training” (TS=193/599, 32.2%): pertains to participants’ interest in training to improve brain health. The barrier was “difficulty in handling technology” (TS=73/599, 12.2%), which refers to the difficulties older adults have in using technologies that are not part of their daily lives.

### Strategies Adopted

Based on these findings in the baseline with participants and stakeholders, we adopted the following targeted strategies with BCTs to increase the level of readiness in each dimension.

1. Knowledge of issue: we shared psychoeducational information through social media about the potential benefits associated with the training (BCT: information about health consequences and problem-solving).
2. Knowledge of efforts: we provided a guide explaining how each cognitive exercise works (BCT: graded tasks, social support).
3. Community climate: we contacted stakeholders and disseminated information about this project (BCT: information about health consequences).
4. Resources: we provided the hardware (computer or tablet) according to the necessity of the participant and provided full support during training and a practical guide on the use of technology (BCT: material incentive and social support [practical]).

### Postintervention and Implementation of the Strategies

Subsequently, we evaluated the readiness levels of both groups following the completion of the training. The results indicated that postintervention participants advanced to the preparation, initiation, and stabilization stages (Table 3).

**Table 3:** Level of readiness postintervention and the application of strategies.

	Group 10 hours training (n=21)			Group 20 hours training (n=20)	
	Median (IQR)	Stage of readiness	Median (IQR)		Stage of readiness
CRM^a^ total	6.0 (5.0-7.0)	Initiation	6.0 (5.0-7.0)		Initiation
Knowledge of issue	5.0 (4.0-6.5)	Preparation	5.0 (4.5-5.5)		Preparation
Knowledge of efforts	5.0 (3.5-6.0)	Preparation	5.0 (4.0-6.0)		Preparation
Community climate	5.0 (4.5-6.5)	Preparation	5.75 (4.5-7.0)		Preparation
Resources	7.0 (7.0-8.0)	Stabilization	7.25 (7.0-8.0)		Stabilization

^a^CRM: Community Readiness Model.

Subsequently, we evaluated whether the readiness levels changed postintervention. The analysis revealed statistically significant increases in all domains of readiness postintervention in both groups, with the exception of resources in the 10-hour training group (Table 4).

**Table 4:** Changes in readiness levels postintervention.

		Baseline, median (IQR)	Postintervention, median (IQR)	Wilcoxon	
				*Z*	*P* value
**Group 10 hours training (n=21)**					
	CRM^a^ total	3.6 (2.2-4.6)	6.0 (5.0-7.0)	–4.01	<.001
	Knowledge of issue	2.0 (3.0-4.0)	5.0 (4.5-5.5)	–3.63	<.001
	Knowledge of efforts	2.0 (1.0-3.0)	5.0 (4.0-6.0)	–3.78	<.001
	Community climate	3.5 (3.0-4.0)	5.0 (4.5-6.5)	–4.02	<.001
	Resources	6.0 (3.0-7.0)	7.0 (7.0-8.0)	–2.78	.51
**Group 20 hours training (n=20)**					
	CRM total	3.7 (2.7-4.3)	6.0 (5.0-7.0)	–3.92	<.001
	Knowledge of issue	2.0 (3.0-4.0)	5.0 (4.5-5.5)	–3.70	<.001
	Knowledge of efforts	2.0 (2.0-3.0)	5.0 (4.0-6.0)	–3.72	<.001
	Community climate	3.5 (3.0-4.0)	5.75 (4.5-7.0)	–3.92	<.001
	Resources	6.0 (4.0-7.0)	7.25 (7.0-8.0)	–3.65	<.001

^a^CRM: Community Readiness Model.

### Facilitators and Barriers Related to Digital Cognitive Training Postintervention

The facilitators post intervention for the 10-hour training group were as follows.

1. Desire to keep training (TS=34/188, 18.1%): refers to participants’ desire to continue training or to start other cognitive stimulation activities after the completion of the training.
2. Team support (TS=46/188, 24.5%): refers to the importance of support and encouragement offered by the research team.
3. Cognitive improvement (TS=58/188, 30.8%): refers to participants’ perception of cognitive improvement after training and sharing information with the community about the benefits of training. The main barrier was “difficulty in handling technology” (50/188, 26.6%). Although the older adults used a computer during training, there were complaints regarding difficulty with access and familiarity in handling technology.

The facilitators postintervention for the 20-hour training group were as follows.

1. Cognitive improvement (TS=26/176, 14.8%): addresses the participants’ perception of cognitive improvement, better performance during the training, and sharing information to the community about the benefits of training.
2. Satisfaction (TS=62/176, 35.2%): refers to the positive experience reported by participants upon completing a new activity.
3. Training challenge (TS=26/176, 14.8%): refers to the participants’ motivation to overcome the challenges of training exercises. The main barrier was “difficulty in handling technology” (TS=62/176, 35.2%): this class reports the difficulty in handling technology and the participants’ need for assistance from a family member during the training.

The qualitative analyses also revealed that participants continued to report difficulties in dealing with technology, even after completing the training, in both the 10-hour and 20-hour training groups. Before the training, older adults reported that they were searching for cognitive stimulation, and postintervention, both groups reported perceived cognitive improvement.

Table 5 complements these findings by providing examples of quotes for each class of facilitators and barriers, offering further insights into the personal experiences of the participants. Additionally, examples of quotes for each dimension from both groups are available in the supplementary material.

**Table 5:** Examples of quotes for each class.

				Quotes
**Baseline**				
	**Facilitators**			
		Interest in the training (193/599, 32.2%)	…*To improve my memory. My attention, I think my attention is very bad, I can’t read books.*	
		Curiosity (100/599, 16.7%)	…*I want to do the training because I found it interesting. I think that curiosity, we are curious to know what it is, what the result is.*	
		Getting results (130/599, 21.7%)	…*Because I want to work my brain to see if I don’t get dementia like my mother did.*	
		Search for cognitive stimulation (103/599, 17.2%)	…*that’s how it is, memorizing, it’s the medication schedule thing, these things, I look for it, I even avoid writing it down so I don’t get complacent.*	
	**Barrier**			
		Difficulty in handling technology (73/599, 12.2%)	…*I have a computer, but I don’t know how to deal with it very well.*	
**Postintervention 10 hours**				
	**Facilitators**			
		Cognitive improvement (58/188, 30.8%)	…*I even noticed that there was some improvement in memory. It’s... to remember things. I thought it improved a lot.*	
		Team support (46/188, 24.5%)	…*I want to thank the girls for their patience with us.*	
		Desire to keep training (34/188, 18.1%)	…*I started to think it was good, I thought it was worth it and that I want to continue if I get the chance.*	
**Postintervention 20 hours**				
	**Facilitators**			
		Cognitive improvement (26/176, 14.8%)	…*I think it improved my mental agility because I came out of the doldrums.*	
		Satisfaction (62/176, 35.2%)	…*I think it was wonderful, it is fantastic to put the brains to work. I’m even recommending my mother and sister to participate.*	
		Training challenge (26/176, 14.8%)	…*I became so obsessed with this task that I kept doing exercises to do better, the reverse. I think I got on with that, doing my silly things, like putting some cream on the toothbrush, that was common thing, a while ago.*	
	**Barrier**			
		Difficulty in handling technology (62/176, 35.2%)	…*I didn’t understand a lot, I think I was doing it all wrong.*	

## Discussion

Our study explored the feasibility of using the CRM to identify barriers and facilitators in implementing digital cognitive training and applying strategies to increase readiness. Older adults were assessed for their initial readiness, which was low but showed significant improvement following targeted strategies and training. Key facilitators included curiosity, motivation to improve cognition, and team support. The main barrier was difficulty with technology, which remained even postintervention. Participants reported cognitive benefits and showed interest in continuing the training. We found that it was feasible to use CRM to identify facilitators and barriers in the implementation of digital cognitive training for older adults, aiming to guide support strategies to enhance readiness levels.

Although our study had a different objective, the observed increase in community readiness for digital cognitive training aligns with findings from institutional delivery services in Ethiopia. While our study addressed individual barriers and motivators, the Ethiopian study used a structured, village-based intervention with education, training, and feedback over 15 months. This highlights CRM’s adaptability to different contexts and strategies [43]. Supporting this, a review study indicated that the CRM was effective in identifying stakeholders, informing them about the intervention, and enhancing community awareness of the issue [44]. Findings suggest that extended exposure to the intervention is associated with higher levels of community readiness, whereas previous research [44,45] has documented annual increases of 0.5 to 1 stage in readiness resulting from sustained intervention efforts.

We successfully recruited a target audience that exceeded the minimum required number of participants. The attrition rate was 7 out of 31 participants (22%) in the group that completed 10 hours of games, with no participants dropping out during the digital cognitive training. In the group that completed 20 hours of training, 1 out of 25 participants (4%) left during the first 10 hours. During the next 10 hours, the attrition rate was 2 out of 24 participants (8%). Attendance was high, with 100% of participants attending all 10 or 20 sessions. Attrition patterns differed between groups, with dropouts in the 10-hour group occurring during the initial game-based activities, which may have been less stimulating than digital cognitive training. In contrast, the 20-hour group had fewer dropouts early on and remained more consistent throughout, possibly due to higher motivation or perceived benefits. External factors, such as health issues and missing data, also contributed to retention differences.

Although CRM has been used in various studies across different fields to promote behavioral changes for better health, such as preventing traumatic brain injury, reducing alcohol use, and supporting older adults, few studies have evaluated readiness before and after the intervention, as we did [23,25,26]. We found that participants had a low level of readiness before the training. However, after implementing the targeted strategies, we observed an increase in readiness, both in the overall CRM score and across its individual dimensions.

The improvement in readiness may be attributed to older adults’ self-perception of benefiting from potential gains associated with training, leading to a greater awareness of the importance of brain training. A systematic review of BCT in computerized cognitive training for older adults described that feedback given by a person about behavioral outcomes can influence adherence and effectiveness of training [11]. In this study, participants received feedback from monitors who accompanied them throughout the sessions on their progress in trained cognitive domains as part of a behavior change strategy, which possibly increased their awareness of the potential benefits obtained from training. The choice to have more personalized feedback from the monitor was based on a systematic review [11] that indicated that when this feedback was provided by a computer, it tended to be less effective.

In terms of the “knowledge of the issue” dimension, participants progressed from the “vague awareness” stage at baseline to the “preparation” stage postintervention. Upon completion, they showed an improved understanding of cognitive exercise and the potential for recovering lost abilities. A qualitative study with older adults with mild cognitive impairment revealed that the computer program used in the study not only enhanced their memory and attention but also assisted in managing daily life challenges from the point of view of the participants [46]. Another study indicated that most older adult participants believed the program was beneficial for their health [47]. Motivators related to cognitive effectiveness, such as the perceived improvement in cognition and tangible evidence of that improvement, were also significant [48]. These findings suggest that as participants recognize the value of digital cognitive training and its impact on their lives, they are more motivated to engage in it.

Participants in this study also demonstrated a significant improvement in the “knowledge of the efforts” dimension. This change was observed as their initial state of “denial” or “resistance” transformed into a state of “preparation.” Over time, participants expressed satisfaction with the potential benefits of cognitive training and recommended it to others, including family and community members. This is further supported by their quotes (Table 4) reflecting satisfaction following the completion of the training.

A study that applied the CRM in the context of childhood obesity prevention also reported significant behavior change by adopting strategies aimed at increasing community readiness. As participants became more aware of the issue and the benefits of healthier behaviors, they actively shared their knowledge with others in the community [49]. In line with these findings, some participants in our study expressed interest in continuing the cognitive training after the program ended, a trend also observed in previous research on cognitive training interventions [47-50]. This suggests that as participants recognize the value of training, they are more likely to seek continued engagement, further reinforcing the importance of fostering sustained motivation and readiness.

The “community climate” dimension, which refers to people’s attitudes toward change—in this case, engaging in digital cognitive training—progressed from the “vague awareness” phase to the “preparation” phase. This improvement can be attributed to the BCT implemented, which kept participants informed about the objectives of cognitive training throughout the process. A Canadian study similarly found that participants who underwent digital cognitive training reported cognitive improvements that were meaningful to their daily lives [51]. Moreover, the absence of adverse effects and the reported cognitive gains likely contributed to an increased sense of self-efficacy among participants, as observed in Edwards et al [52] study on the outcomes of digital cognitive training programs.

Before the training, the “resources” dimension was already at the “initiation” stage, as access to technological devices was a prerequisite for participation. Despite a significant improvement in this dimension, participants frequently reported difficulties in handling technology both at baseline and at postintervention. This barrier, also highlighted by stakeholders during baseline assessments, reflects the digital exclusion often faced by older adults, who are less likely to use computers and the internet [53-55]. Similarly, other studies have noted limited access to devices and a lack of proficiency in using digital tools among older adults, particularly computers [54]. To address this issue, we recommend that future studies incorporate digital inclusion initiatives prior to formal training sessions. Such initiatives could help familiarize older adults with technology, build their confidence, and improve their proficiency. Evidence from recent research, such as Djabelkhir et al [56], supports this approach, showing that pretraining technology familiarization enhances participants’ skills, fosters positive attitudes toward training, and boosts self-efficacy, ultimately leading to more successful digital literacy outcomes.

In our study, stakeholders showed a low level of readiness, contrasting with research that highlights strong professional support for digital cognitive programs and their role in preserving cognitive function among older adults [56]. Stakeholders in our study faced challenges in helping participants consistently integrate training into their routines, primarily due to limited familiarity with technological resources. Addressing this issue calls for collaborative efforts to enhance stakeholders’ understanding of digital cognitive training and to develop strategies that effectively promote older adults’ engagement with these tools.

The main facilitators identified in the baseline were related to the interest in the training, getting results, searching for cognitive stimulation, and curiosity. The facilitators identified in the postintervention analysis for the group that did 10 hours of training were related to cognitive improvement, team support, and the desire to continue training. While, for the group that completed 20 hours, the facilitators were related to cognitive improvement, satisfaction, and training challenge. Notably, the “training challenge” reported by the 20-hour group highlights the importance of programs that adapt to an individual’s progress throughout the intervention [10].

In this study, participants were not compensated for their involvement, reflecting real-world conditions where individuals typically participate without financial incentives. Their voluntary engagement created a naturalistic setting, enhancing the generalizability of the findings to everyday contexts. This study has some limitations. Although we tried to diversify the sample for data collection, the criteria for participation in this study included the participant having access to a computer and the internet. As a consequence, we have a selected sample, which does not allow the generalization of these findings to populations with different characteristics. Another limitation of this study concerns the fact that we did not interview stakeholders after the participants completed the digital cognitive training, which prevented us from determining whether there were similar changes in readiness. Finally, this feasibility study was designed to establish the viability, and not to demonstrate the effectiveness of the use of CRM to increase readiness in older adults [53]. Thus, the current findings of an increase in readiness should be interpreted as exploratory and need to be confirmed.

We propose several recommendations for future research aimed at increasing readiness for digital cognitive training among older adults: (1) disseminate psychoeducational content on digital cognitive training and its associated benefits; (2) incorporate digital literacy training before cognitive training, especially for older adults who have difficulty handling technology; (3) provide feedback on training performance and the cognitive domains being trained; and (4) disseminate study findings not only at scientific events but also through mainstream media.

In conclusion, CRM is a feasible tool for identifying facilitators and barriers that can inform strategies to enhance readiness for digital cognitive training in older adults. Future studies should assess the effectiveness of applying CRM to improve readiness and examine whether higher levels of readiness are associated with greater engagement and cognitive improvements following digital cognitive training in older adults. Notably, while CRM was selected in this study due to its comprehensive framework encompassing multiple dimensions, key stakeholders, and the population of older adults, alternative models addressing behavior change may also contribute to the successful implementation of digital cognitive training programs. Therefore, further research exploring diverse theoretical frameworks is encouraged to optimize cognitive training interventions for older adults.

## Abbreviations

BCT: behavior change technique
CRM: Community Readiness Model
TS: text segment
